# Nurses’ Perceptions of Communication in an Oncology Hospital Care: A Qualitative Study

**DOI:** 10.3390/healthcare14010121

**Published:** 2026-01-04

**Authors:** Lara Guariglia, Maria Condoleo, Giovanna D’antonio, Simona Molinaro, Tatiana Bolgeo, Francesca Gambalunga, Fabrizio Petrone, Anita Caruso, Laura Iacorossi

**Affiliations:** 1 Psychology Unit, IRCCS “Regina Elena” National Cancer Institute, Via Elio Chianesi, 53, 00144 Rome, Italy; lara.guariglia@ifo.it (L.G.); maria.condoleo@ifo.it (M.C.); giovanna.dantonio@ifo.it (G.D.); anita.caruso@ifo.it (A.C.); 2 Nursing Research Unit IFO, IRCCS Regina Elena National Cancer Institute, Via Elio Chianesi, 53, 00144 Rome, Italy; 3 Department of Research, Training and Innovation, Azienda Ospedaliera Nazionale S.S. Antonio e Biagio e C. Arrigo, 15121 Alessandria, Italy; tbolgeo@ospedale.al.it; 4 Professional Health Care Services Department, University Hospital “Policlinico Umberto I”, 00161 Rome, Italy; f.gambalunga@policlinicoumberto1.it; 5 Health Professions Unit, IRCCS “Regina Elena” National Cancer Institute, Via Elio Chianesi, 53, 00144 Rome, Italy; fabrizio.petrone@ifo.it; 6 Department of Life, Health and Health Professions Sciences, Link Campus University, 00165 Rome, Italy; l.iacorossi@unilink.it

**Keywords:** nursing competencies, communication skills, oncology, patients, quality of care, caring, hospital setting

## Abstract

**Highlights:**

**What are the main findings?**

- Nurses identify communicative competence as a core professional skill essential for fostering trust, addressing psychosocial needs, and ensuring high-quality, patient-centred oncology care.
- Significant barriers—such as staff shortages, workloads, emotional distress, cultural differences, and insufficient training in advanced communication competencies—limit nurses’ ability to apply these skills effectively.

**What are the implications of the main findings?**

- Strengthening communication skills through targeted training and multidisciplinary support may improve both care quality and nurse well-being.
- Organizational models that support adequate time and resources for nurse–patient communication may enhance patient-centred oncology care and contribute to the broader advancement of contemporary nursing competencies.

**Abstract:**

**Background/Objectives**: In the context of evolving healthcare systems, effective communication represents a fundamental skill for ensuring quality care and addressing the psychosocial needs of oncology patients. In line with the new challenges of nursing education, this study explores communication between nurses and oncology patients, analyzing facilitating and hindering factors from the nurses’ perspective within the hospital setting. **Methods**: A descriptive qualitative study was conducted using one-on-one semi-structured interviews. The interviews lasted from 15 to 30 min. The study population consisted of nurses working in the Medical Oncology units of the Regina Elena Institute in Rome (IRE). Data were analyzed using the Framework Analysis method by Ritchie and Spencer. **Results**: The sample consisted of 20 nurses with an average age of 33.5 years. Six main themes emerged: communication as the pillar of the care relationship between technical and human aspects, the need for a balance between closeness and personal protection, the influence of language and personalized approaches on communication, the stimulation of specific training needs, and barriers to nursing communication. **Conclusions**: Nurses recognize communication as an integral part of the care process and as a key competency for addressing the complex needs of oncology patients. However, inadequate training, time constraints, and staff shortages represent significant barriers, highlighting the need to invest in specific training programs and organizational strategies to improve the quality of care.

## 1. Introduction

Current clinical standards advocate a comprehensive care model for oncology patients that ensures continuity in relationships, information, and management [1]. Within this model, particular attention is given to the relational aspect of the care process and the importance of effective communication with patients. Recent studies demonstrate that, in the healthcare setting, effective nurse–patient communication improves symptom management and pain control and reduces emotional distress [2,3], enhances patient participation in treatment decisions [4], and increases adherence and satisfaction with care [5]. These findings further support the central role of communication in improving care outcomes in oncology setting. In the comprehensive care model, the nurse is not only a provider of interventions but also a key professional ensuring relational, informational, and managerial continuity, capable of integrating clinical, communication, and relational skills [1]. In hospital oncology care, nurses play a central role in the communication process as they have more frequent and continuous interactions with patients and their families than other healthcare professionals. Nurse–patient communication is an integral part of daily care; this process may include conveying personalized information, investigating the patient’s psychosocial context, and establishing a therapeutic alliance, all of which contribute to improved care outcomes [6,7]. The nurse can support the patient in explaining diagnostic procedures or treatments, in recognizing and reassuring fears, in offering emotional support to family members, and in coordinating between different health services [1]. In this perspective, communication represents a core nursing competence necessary for addressing patients’ complex needs.

In addition to direct communication, nurses often serve as intermediaries between patients, their families, and physicians, as highlighted in the literature [8]. However, fulfilling this role requires not only adequate preparation and professional training but also an organizational and management structure that supports communication as an integral component of the care process [9]. This underscores the relevance of developing communication-related nursing competencies within the clinical setting.

Evidence reports that nurses face various communication challenges, particularly due to limited experience in managing complex situations, which can lead to feelings of inadequacy and affect their professional self-perception [10]. Additionally, time constraints and limited structural resources often cause nurses to focus more on patients’ physical needs rather than their psychosocial needs [11]. Internationally, nursing education programs vary and often lack specific training in communication skills, which may hinder the development of adequate communicative competencies in clinical practice. This gap may lead to overlooked patient cues, potentially compromising the quality of the nurse–patient relationship [12].

Effective communication requires essential elements such as genuineness, warmth, and empathy, which are crucial for building a strong care relationship [13]. Literature presents mixed findings regarding patient satisfaction with nurse communication. Some studies highlight a discrepancy between nurses’ communication approaches and patients’ hospitalization experiences, reflecting organizational priorities rather than individual patient needs [1,14]. Conversely, other studies report high patient satisfaction, both in terms of time dedicated to them and the completeness of the information provided [15].

A study on oncology patients hospitalized in the Medical Oncology units of the Regina Elena Institute (IRE) found that nurse–patient communication is generally considered satisfactory. A confidential communication style, direct language, and caring attitudes emerged as facilitators of communication, while lack of communication between medical and nursing staff, staff shortages, and limited time were identified as the main barriers [16]. These factors reinforce the importance of strengthening communicative competencies to support high-quality care.

In light of these findings, we aimed to further explore nurses’ direct perceptions to assess the extent to which clinical practice within inpatient oncology care aligns with the currently promoted comprehensive care model. Specifically, this study sought to: (i) examine, through nurses’ firsthand experiences, the quality and nature of communication with patients; (ii) identify perceived facilitating factors that support effective communication with patients and their families; and (iii) explore organizational, professional, and contextual barriers that hinder communication in daily clinical practice.

## 2. Materials and Methods

### 2.1. Design

This study is a descriptive, inductive, single-center qualitative research, structured according to the Consolidated Criteria for Reporting Qualitative Research (COREQ) to ensure methodological rigor and transparency [17,18].

### 2.2. Study Setting and Sample

A purposive sampling [19] was employed to recruit nurses working in the Medical Oncology Units at the IRE in Rome. The inclusion of nurses from these units ensured direct experience with the phenomenon under investigation, allowing for a comprehensive understanding of communication dynamics within the hospital setting. The inclusion criteria required that participants were nurses working in the Medical Oncology Units at IRE, with at least six months of experience in oncology care; participants also needed to be available for interviews to provide detailed qualitative insights into their professional experiences and sign written informed consent to confirm their voluntary participation in the study and their adherence to ethical research principles. The sampling strategy and inclusion criteria were defined a priori to ensure coherence with the study aims and methodological rigor. The sample size was determined based on data saturation, following the approach of Guest et al. [20]. Achieving saturation ensured the robustness of the qualitative analysis and strengthened the reliability of the findings [19,20].

### 2.3. Instruments

The study employed a semi-structured interview approach, developed according to the researchers’ expertise and relevant literature [21,22]. The formulation of the questions was based on a phenomenological theoretical approach and on the use of Reflexive Thematic Analysis (RTA), which involved the use of open-ended questions, minimal researcher interference in predetermining categories, and the facilitation of participants’ meaning-making [23]. A panel of experts in oncology and qualitative research then reviewed the questions to ensure clarity, relevance, and effectiveness in eliciting meaningful responses [23]. To ensure the reliability of the instrument, a pilot test was conducted with two participants, confirming the clarity and effectiveness of the questions and allowing for necessary refinements before full implementation [24]. The pilot test involved two nurses working in the same institution but in clinical units different from those included in the main study, each with at least two years of oncology experience. Their feedback focused on the clarity and sequencing of the questions. They suggested minor linguistic simplifications, a slight reordering of the initial items to facilitate narrative flow, and the inclusion of optional prompts to encourage the emergence of concrete communication episodes. Based on their feedback, small adjustments were made to the wording of the guide and additional probes were incorporated, without requiring substantial changes to its overall structure. The interview guide was developed in Italian and used in this language during data collection. For publication, it was translated into English through a two-step process involving translation by a bilingual researcher and independent review for semantic accuracy. The full interview guide is provided as Supplementary Material (File S2). The guiding questions developed through this process enabled participants to describe their personal experiences regarding communication with patients, reflecting on both facilitating and hindering factors. Although the interview guide consisted of broad, open-ended questions, the interviewers used their clinical experience to explore, through in-depth questions on the emerging content, key dimensions relevant to communication in the oncology field. This approach ensured that the interviews elicited rich, experience-based narratives rather than general opinions. A final question was included to encourage participants to provide practical suggestions for improving nurse–patient communication (Table 1). The interviews were conducted by experienced oncology nurses trained in qualitative interviewing, which supported reflexivity and consistency throughout the data collection process. In addition, a socio-demographic data collection form was used to complement the interviews and gather essential contextual information about the participants. This approach ensured the collection of relevant data to contextualize the experiences reported in the interviews without including clinical or other personal details [22].

### 2.4. Data Collection

The semi-structured interviews took place between October 2023 and January 2024 in private and comfortable rooms in the Medical Oncology Units of the IRE in Rome to ensure uninterrupted discussions. Although situated inside the clinical environment, these rooms were specifically designated for confidential conversations and were temporarily reserved for the study to minimize clinical interruptions. To further reduce the likelihood of disruptions, interview slots were scheduled in coordination with unit staff during periods of lower clinical activity. The interviews were conducted by two psycho-oncologists, responsible for recruiting participants, explaining the study’s objectives, and overseeing data collection. Both interviewers worked in the same institution but not in the same clinical unit, which helped ensure that role-related power dynamics and social desirability were appropriately managed. Their clinical experience facilitated the development of rapport with participants and supported a reflective awareness of potential preconceptions. Nurses were invited to carefully read and sign the informed consent form to ensure an informed choice regarding their participation. Data collection continued until data saturation was reached [25]. Saturation was operationalized as the point at which no new codes, categories, or relevant conceptual insights emerged during the iterative analysis of consecutive interviews. After approximately the twelfth interview, the research team observed that the coding framework had stabilized and that subsequent interviews only confirmed previously identified patterns without adding new thematic elements. To enhance methodological transparency, the researchers also actively searched for deviant or negative cases, examining whether any interview contradicted or substantially challenged the emerging themes [26,27]. No such cases were identified, further supporting the assessment that saturation had been achieved. This ensured analytical rigor and strengthened the reliability of the study. Throughout the interviews, the psycho-oncologists maintained flexibility, exploring emerging topics and asking follow-up questions to collect rich, in-depth qualitative data [23]. Each interview was conducted individually, audio-recorded, and later transcribed using the “smooth verbatim transcription” method, which preserves participants’ words while adjusting dialectal expressions for clarity [23].

### 2.5. Data Analysis

The study employed Framework Analysis to systematically examine the data, ensuring transparency and analytical rigor throughout the process [28,29]. This approach allowed researchers to explore participants’ experiences in depth while maintaining structured control over the interpretation process, thereby enhancing the credibility and reliability of the findings.

The analysis, following Ritchie and Spencer’s method [30], was carried out in five stages:

1. Familiarization—Two researchers listened to and transcribed the interviews, reviewing each transcript multiple times to identify key ideas and recurring themes.
2. Thematic framework identification—Emerging themes were assessed in relation to the study’s objectives and the questions in the interview guide to develop an analytical framework.
3. Indexing—Data segments were systematically categorized to ensure accuracy and facilitate a deeper examination of patterns within the responses.
4. Charting—Summaries of the categorized data were organized under thematic headings to provide a structured overview of the findings.
5. Mapping and interpretation—Visual tools, including graphics, were employed to trace connections between themes and categories, offering a comprehensive understanding of the phenomenon (Table 2).

All analyses were conducted using NVIVO v.12 software.

### 2.6. Reliability and Validity

To enhance the credibility of the findings, multiple strategies were adopted in line with Lincoln and Guba’s paradigm [31]. Triangulation was employed through continuous sharing of results among researchers to ensure internal consistency. The research process was thoroughly documented to enable future replication and strengthen reliability. A cross-review technique was implemented, allowing independent researchers to compare emerging themes with the original transcripts and mitigate potential biases. Additionally, detailed participant descriptions were provided to support transferability, helping readers assess the applicability of the findings in different contexts. These procedures were consistent with the Standards for Reporting Qualitative Research (SRQR) [32], supporting the credibility, dependability, and transparency of the analytic process.

### 2.7. Ethical Considerations

This study was conducted in accordance with Good Clinical Practice (GCP) guidelines, in compliance with the requirements of regulatory authorities and key European and national regulations governing ethical research practices. Approval was obtained from the Lazio District 5 Territorial Ethics Committee, as documented in Verbal Extract no. 3 of 5 September 2023, and registered under Trial Register Experiments No. 34/IRE/23. The study adhered to ethical principles, ensuring transparency, participant protection, and compliance with established regulatory standards.

## 3. Results

### 3.1. The Characteristics of the Participants

The study included 20 nurses, who were interviewed for an average of 30 min each. Demographic data for nurses are included in Table 3.

### 3.2. Emerged Themes

The themes emerging from the analysis are presented below, accompanied by meaningful quotations from the participants that highlight key findings.

#### 3.2.1. Communication as the Pillar of the Care Relationship Between Technical Aspects and Humanity

In nursing practice, communication is more than just a tool for interaction; it represents the very essence of the care relationship. Through dialogue, nurses not only establish trusting relationships with patients but also directly influence their therapeutic journey and the quality of care. Nurses recognize communication as a means of building a functional alliance with patients, that is, a collaborative relationship, based on shared objectives, which is supporting both the implementation of the care and treatment plan and the fulfillment of psychosocial needs. For this reason, it is considered one of the fundamental elements of the profession.

“*In my opinion, communication is the basis of everything, because that is where the therapeutic plan for a patient originates or begins.*” (I6) “*So far, I would define communication as a fundamental aspect in establishing the care relationship, which is one of the cornerstones of nursing care.*” (I19) “*Communication is crucial—of course, it is important in all fields—but in oncology, it is almost everything…. if not everything.*” (I14)

The central role of communication is also reflected in how nurses act as mediators between patients and the healthcare system:

“*The nurse is really seen as an emotional outlet… In front of a doctor, the patient—whether due to anxiety or lack of confidence—freezes up… In the end, we nurses listen to what the patient has to say, for better or worse.*” (I11) “*Communication must be welcoming; when the patients know how you operate within the ward, they feel more open, even to receiving care.*” (I16)

Thus, communication emerges as a key element that balances the technical aspect and the humanity of nursing care, enabling the creation of an effective, patient-centered care environment.

#### 3.2.2. Communication Requires a Balance Between Closeness and Personal Protection

The relationship with patients requires energy and significant emotional effort. Nurses strive to establish the right boundary of involvement in their interactions, especially when dealing with young patients and their families. The desire to be close to patients and support them in their most difficult moments often clashes with the need to maintain emotional distance to preserve their personal balance. Sustained emotional involvement exposes professionals to the risk of emotional overload, compassion fatigue, and burnout. As a result, healthcare professionals frequently engage in a continuous process of boundary negotiation, striving to remain empathically engaged while simultaneously maintaining a degree of emotional distance necessary to preserve their own psychological well-being.

“*I wake up at night thinking about that patient… I can’t put up a barrier, so I take it home with me… I try hard not to talk about it with my family… but I still think about it.*” (I11) “*I kept thinking about the patient even after they were discharged…we also get emotionally attached.*” (I14)

This need to protect oneself emotionally becomes even more apparent when dealing with particularly vulnerable patients:

“*Mostly with very young patients—those who could be my own children—where, most of the time, the staff has to put on a mask to be able to communicate with them.*” (I11) “*In many cases, we care for underage patients… There’s a stronger emotional investment… Sometimes, I bring it home with me.*” (I13)

The ability to set boundaries while maintaining empathy is therefore a fundamental skill for healthcare professionals, particularly in delicate areas such as oncology.

#### 3.2.3. The Care of Language and a Personalized Approach Influence Communication

Nurses are aware of the most effective and appropriate communication methods, both in terms of verbal aspects (clarity of vocabulary), paraverbal aspects (tone of voice), and relational dynamics (understanding the subjectivity of others, recognizing nonverbal cues in communication, and interpreting the most useful interactive role).

Clarity, tone of voice, and a personalized approach

“*Clear communication certainly helps guide the patient along a more straightforward treatment journey…*” (I14) “*Maintaining a low tone… when communication is too abrupt, it doesn’t allow for proper interaction…*” (I5)

Personalizing the approach is considered crucial for ensuring effective interaction with patients:

“*Understanding whether a person needs a joke or a firmer approach… or if, at that moment, they simply need to talk.…*” (I3) “*Avoid standardizing patients… rather than following a generic guideline for everyone, it’s important to find a different approach for each individual…*” (I15)

In addition, nurses recognize the importance of adapting their language and approach to the patient’s age and family context. They are aware that communication is not a one-size-fits-all process; rather, it requires sensitivity to developmental stage, generational expectations, health literacy, and the role played by family members in decision-making and care:

“*With adults, the language is different from what you use with children… the attitude changes…*” (I1) “*Age also plays a role… elderly men are all grandfathers, and elderly women are all grandmothers…*” (I20)

Nonverbal signals and gestures in communication

In addition to words, nonverbal communication plays a key role in the care relationship. Nurses understand that patients carefully observe their attitudes, expressions, and gestures to gauge their level of trust.

“*Verbal communication is important, but so is nonverbal—sometimes, even a simple caress on the hand matters a lot.*” (I12) “*Physical contact is a form of nonverbal communication that, in my opinion, patients feel much more deeply than words.*” (I16)

Nurses also acknowledge the importance of managing their own expressiveness, as patients are keen observers:

“*Patients are real observers; when we enter a room, they watch how we behave…*” (I6) “*You always have to be mindful of everything you do, especially with oncology patients.*” (I16)

Effective communication in nursing goes beyond words—it requires a balance of clarity, tone, personalization, and an awareness of nonverbal cues. By tailoring their approach to each patient’s needs and recognizing the impact of gestures and expressions, nurses foster trust and connection, ultimately enhancing the quality of care. Investing in communication skills ensures that every interaction supports not only clinical outcomes but also the emotional well-being of patients.

#### 3.2.4. Communication Activates the Support of Specialized Figures

Nurses recognize the need for a structured and integrated organization to address the therapeutic and psychosocial needs of patients. They emphasize how the role of the case manager and the presence of a psychologist could significantly improve the quality of care and treatment.

Nursing communication, when incorporated into this broader system, would benefit from the contribution of specialized professionals, fostering an integrated team that enhances both therapeutic care and emotional support for patients and healthcare workers alike.

“*The role of the case manager… is not widely used… but it could really be a great help.*” (I18) “*Maybe a psycho-oncologist could come to the wards and ask us nurses, ‘Which patients do you think would need this support?’”* (I16) “*We need a space to let go of all these emotions and experiences.*” (I5)

This highlights the importance of professional figures who not only assist patients but also provide mental and emotional support for nurses, ensuring better care and well-being within the healthcare environment.

#### 3.2.5. Communication Stimulates the Need for Specific Training

Nurses emphasize the need for dedicated courses to improve communication skills, in both university education and continuing professional training. Interaction with patients requires specific skills that cannot simply rely on spontaneous personal abilities, rather they must be professionally structured.

“*In my opinion, nurses should receive specific training on how to interact with children and their parents…*” (I1) “*Perhaps pedagogy should be incorporated into the third year of nursing studies…*” (I16) “*Courses dedicated to nurses could enhance communication…*” (I11)

Investing in formal communication training ensures that nurses can engage with patients in a way that is effective, empathetic, and tailored to individual needs. By incorporating structured learning pathways, healthcare organizations can strengthen both patient care and professional development, enhancing the overall quality of interactions within the medical field.

#### 3.2.6. Obstacles to Nursing Communication

The Role of Emotions and Cultural Differences

The quality of communication between nurses and patients can be influenced by various emotional and cultural factors. Anger, fear, and depression can hinder interaction, making dialogue difficult.

“*Sometimes they create distance because many arrive visibly upset…*” (I15) “*They have taken their illness badly and blame the whole world—including us…*” (I20)

A patient’s level of education and cultural background also play a significant role: a well-informed and open-minded patient may have a better understanding of healthcare information, whereas those with more complex life experiences may struggle to accept the therapeutic process.

“*Patients are different… it also depends on their family background—if they have support, they deal with the issue differently…*” (I6) “*Cultural background plays a significant role—not in terms of level of education but rather open-mindedness.*” (I18)

*Barriers Related to Illness and Understanding Information*

Communication about the illness may be hindered by patients’ defense mechanisms, the complexity of medical information, family interference, language barriers, or even cognitive impairments. As with communication with minors, end-of-life situations present additional challenges due to a lack of specific training on relational management during this stage.

“*They don’t process the information because there is so much of it and because at first they deny it…*” (I8) “*Language barriers… physical barriers… family members often interfere with communication… privacy issues…*” (I18) “*For me, talking to terminally ill patients is difficult because I don’t really know what to say…*” (I12)

Lack of Clarity and Difficulty in Communicating Bad News

One’s personal background and attitude toward work and patients can all interfere with communication. Clear communication is essential, as patients—due to their crisis condition—may misinterpret the intended message.

“*Even though you think you were clear, the other person might interpret something completely different…*” (I17) “*There is often a lack of clarity, which is a major barrier. Sometimes, bad news is sugarcoated, making it harder to accept…*” (I18) “*It depends a lot on personality—if someone approaches the patient poorly, in a rude or dismissive manner, communication simply won’t happen…*” (I4)

Workload and Lack of Time

Nurses recognize that effective communication requires time. However, tight schedules, staff shortages, and high workloads often prevent nurses from fully engaging in patient dialogue.

“*If there’s no time to exchange information, genuine communication is impossible…*” (I17) “*Being in a hurry does not help communication with patients…*” (I19) “*Unfortunately, there are reasons why we struggle—with time and workload being the biggest ones.*” (I16)

Nursing communication is a complex process, influenced by multiple factors that can hinder patient interaction. Emotional and cultural barriers, challenges in managing medical information, lack of clarity, intense workloads, and staff shortages make communication a daily struggle for nurses. Addressing these obstacles requires sensitivity, adaptability, and effective strategies, as well as continuous training and organizational resources. Investing in the improvement in communication strengthens the quality of patient care and ensures greater understanding and better support for patients and their families.

## 4. Discussion

The aim of this study was to gather the experiences reported by nurses working in the medical oncology units of the IRE regarding communication with hospitalized patients and to explore the factors perceived as influencing this process, either facilitating or hindering it. The results revealed that nurses recognize communication with patients as an integral part of the care process, serving as a means to establish a functional alliance for both the implementation of the care and treatment plan and the fulfillment of psychosocial needs. This finding may be explained by the average age of the sample and it appears to align with the evolving care models and the specific training in nursing sciences, which considers nurse–patient interaction essential for fostering patient participation in healthcare. Nurses report that affectivity and empathy are essential dimensions in their relationship with patients. However, they also acknowledge the importance of establishing appropriate boundaries in their involvement, particularly when interacting with young patients and their families. These interpretations should be considered in light of the study’s single-site design and reliance on self-reported experiences, which may limit the breadth of perspectives captured. Although this result could be influenced by social desirability, considering the normative value attributed to empathy in healthcare professions, the previous literature confirms that the specific characteristics of oncology wards, such as prolonged hospitalizations and continuity of care, can foster more lasting and empathic relationships with patients [33,34]. These same elements, however, can introduce relational difficulties: several studies report that emotional burden, exposure to suffering and end-of-life situations can complicate interactions and increase the risk of emotional fatigue [34]. A systematic review examining the relationship between empathy and psychological distress found that empathy may be associated with the risk of burnout [35]; other studies show that both high and low levels of empathy are associated with higher rates of depression, while moderate levels of empathy offer the greatest protection [36]. If adequately supported, nurses’ empathic behavior increases work motivation, facilitates the acquisition of more accurate information about the patient, allows a better interpretation of needs, improves problem management and leads to more effective treatments [37,38]. Nurses make reference to verbal and nonverbal communication skills and strategies, demonstrating awareness that emotional meanings can be embedded in informational signals, which arise both from patients and nurses, in a circular process. These themes are consistent with prior qualitative research conducted in different oncology hospital contexts [11] but they must be interpreted with caution because the absence of direct observational data prevents assessing how these communication processes unfold in real-time interactions. They also recognize their role as intermediaries in communicating with family members [8], emphasizing the importance of a communication network that extends beyond the patient–nurse relationship. Nevertheless, a tension emerges between ideal principles and practical realities in patient care. Nurses acknowledge the need for a structured and integrated organization that addresses therapeutic and psychosocial needs, emphasizing the importance of specialized roles (case managers), adequate training models, and workload management that includes time devoted to relationships and communication. Despite these individual and organizational challenges, the literature tells us that nurses’ communication practices do not develop in isolation but are shaped by the broader multidisciplinary environment in which they work. In oncology units, observing how other professionals interact with patients helps nurses refine and align their own communication strategies, ensuring coherent and patient-centered messages across the team [39]. The experiences reported align with existing literature [11] and reinforce the need for planning healthcare services in accordance with modern patient-centered care models [40]. Factors such as feelings of incompetence, difficulty in delivering necessary care, and the lack of an adequate support system contribute to moral distress among nurses [41]. Working conditions—including staff shortages, lack of privacy, and high daily workload—are considered major obstacles to effective patient communication. This is consistent with the literature, which identifies organizational factors such as heavy workloads, insufficient staffing, and time pressure as barriers that limit nurses’ ability to engage in meaningful dialogue with patients [42]. Additional barriers have been identified, including inadequate training in communication competencies, insufficient clarity regarding professional roles, emotional challenges in addressing sensitive issues such as prognosis, suffering, and death, as well as organizational and cultural obstacles associated with rigid hierarchies and limited emphasis on patient-centered communication [34]. However, the way in which such barriers are experienced may also reflect the specific organizational culture of the study setting, which limits the transferability of these findings to different contexts. The emotions expressed by patients are considered barriers to communication. Nurses recognize the difficulty of managing some patients’ psychological conditions, such as depression or anger, due to insufficient training. Oncology patients often express psychological distress and seek informational and emotional support, sometimes in dysfunctional ways [43]. Nurses fear their inability to meet patient needs, which can lead to avoidance behaviors [44,45]. This finding appears particularly relevant in oncology, where prolonged hospitalization and continuity of care tend to foster more empathetic and emotionally invested relationships [33]. Thus, while such empathy can enhance the therapeutic alliance, it may also heighten nurses’ sense of responsibility and vulnerability, making the perceived risk of failing to meet patient expectations even more distressing. Communicating information about the disease can be hindered by patient defense mechanisms, such as denial of the illness, the amount and complexity of medical information, barriers imposed by family members, language differences, or patient impairments. Similar challenges are observed when communicating with minors and in end-of-life care, where nurses report difficulties due to a lack of specialized relational training for these situations. These findings align with the previous literature [7]. Finally, additional barriers to communication are linked to personal traits, including individual personality characteristics, work attitude, and general predisposition toward relationships. Literature identifies key factors that support nursing practice, such as training, leadership, adaptability, and motivation. Of particular relevance to oncology nurses is “adaptability”, as it reflects the complexity of modern oncology care [46]. The findings of this study align with patient-reported experiences [16]: the facilitators and barriers to communication are consistent across both groups. In summary, this study highlights nurses’ strong awareness of the principles and skills required for effective patient communication, while also recognizing the need for structured organizational support to address therapeutic and psychosocial needs, particularly in terms of time, resources, and specialized training. These conclusions should be interpreted with caution, given the limited demographic diversity of the sample and the potential influence of social desirability in self-reported accounts.

### 4.1. Practice Implications

The recognition of communication as a cornerstone of the care relationship highlights the need to consider nurse–patient communication as a core clinical skill, on a par with technical competencies. It is therefore necessary to integrate communication objectives into care plans and to value the time dedicated to the relationship as time for care, rather than as an ancillary activity. The lack of specific communication training calls for structured implementation pathways for communication in oncology, both in graduate programs and in continuing education, including modules dedicated to complex communication situations (managing intense emotions, communicating with minors and families, and end-of-life care). Finally, it is important to introduce structured emotional support spaces for nurses, strengthen multidisciplinary teamwork, and enhance coordination roles (e.g., case managers) to improve continuity of care and current care models.

### 4.2. Strengths and Limitations

This study provides an in-depth understanding of patients’ experiences, which represents a key strength of qualitative approaches. However, the interpretive and context-dependent nature of this methodology limits the generalizability of the findings and makes full replicability challenging. The single-center design, conducted within one Roman healthcare setting, further restricts the transferability of the results to contexts with different organizational or cultural characteristics. The study results demonstrate a combination of context-specific elements and widely transferable dimensions. The contextual elements are that IRCCS oncology hospitals are highly complex institutions, with medium- to long-term hospitalizations and a high level of information and decision-making. Some organizational and multidisciplinary integration methods are not structurally present as in other contexts [47]. Furthermore, the strong involvement of families in the treatment process is a characteristic of Italian culture. The thematic clusters relating to the centrality of communication, the management of empathic balance, the personalization of language, training needs, and organizational barriers are consistent with the international literature, suggesting a good transferability of the findings to other hospital-based oncology settings. To overcome these limitations, future research should involve multiple sites and adopt mixed-methods designs, integrating qualitative and quantitative data to capture a broader range of experiences and validate the findings across more heterogeneous populations.

### 4.3. Recommendations for Future Studies

In light of these findings, it is essential to further investigate the impact of nursing education on communication skills. Future studies could examine how academic preparation and professional experience influence nurses’ ability to interact with oncology patients, assessing the effectiveness of targeted training programs. Furthermore, exploring strategies to improve interdisciplinary communication between nurses and other healthcare professionals could help create a more integrated and collaborative work environment. Future research could also focus on developing organizational models that allow for more effective management of time dedicated to communication, reducing moral distress among nurses, and ensuring more comprehensive, patient-centered care. These approaches underline the importance of developing and strengthening communicative competencies within nursing education and clinical practice.

## 5. Conclusions

This study reports on nurses’ experiences communicating with hospitalized oncology patients in medical oncology units. Effective communication between nurses and oncology patients, supported by nurses’ specific competencies, is a crucial element in ensuring adequate treatment, high-quality care, and patient support throughout the care process. Clear, empathetic, and skillfully executed communication can significantly enhance patient well-being during hospitalization. However, critical challenges—such as interaction with young patients and communicating during the terminal phase of the illness, staff shortages, workload pressures, and organizational constraints, hinder the effectiveness of this process.

## Figures and Tables

**Table 1:** Guiding questions for interviews.

Could you describe your experience with patient communication in the ward? If positive: What elements have helped facilitate communication? What aspects, if any, could still pose challenges? If negative: What factors have hindered communication? What elements could contribute to improving it? Do you have any recommendations for enhancing communication between nursing staff and patients?

**Table 2:** Example of the coding process in inductive analysis.

No. Interview	Codes	Theme
6	It is the basis of everything	Communication is the pillar of the care relationship between technical aspects and humanity
11	The nurse is seen as an emotional outlet… we listen to what the patient has to say	
11	Patients find someone they can talk to, someone they can share their problems with	
14	It is essential to care	
14	Communication is almost everything… if not everything	
16	Communication must be welcoming	
17	Communication is an important part of care	
18	It is the foundation of the relationship between nurse and patient	
19	A fundamental aspect of establishing the care relationship, which is one of the cornerstones of nursing care	

**Table 3:** Nurses Data.

Nurse Code	Age	Gender	Educational Level	Start Working	Start Working in Medical Oncology Units
1	44	F	Graduate, I, II Level Master’s	2002	2022
2	23	F	Graduate, I level Master	2022	2022
3	42	M	Graduate, I level Master	2004	2004
4	27	F	Graduate, I level Master	2019	2021
5	40	F	Graduate	2008	2021
6	51	F	Upper Secondary	1999	2004
7	28	F	Graduate	2019	2022
8	30	M	Graduate	2021	2021
9	32	M	Graduate	2014	2018
10	26	M	Graduate	2016	2021
11	43	F	Graduate	2017	2020
12	23	F	Graduate	2022	2023
13	31	M	Graduate	2017	2023
14	30	F	Graduate	2023	2023
15	28	F	Graduate, I level Master	2018	2023
16	34	F	Graduate	2023	2023
17	57	M	Graduate	1997	2004
18	27	F	Graduate	2020	2020
19	27	M	Graduate	2022	2023
20	27	F	Graduate	2020	2020

## Data Availability

The data that support the findings of this study are openly available at: https://gbox.garr.it/garrbox/s/Vv2T997M5d8daNe (accessed on 19 June 2025).

## Supplementary Materials

The following supporting information can be downloaded at: https://www.mdpi.com/article/10.3390/healthcare14010121/s1, File S1. COREQ (Consolidated criteria for Reporting Qualitative research) checklist. File S2. Full Interview Guide.

## Abbreviations

The following abbreviations are used in this manuscript:

IRE	Regina Elena Institute
COREQ	Consolidated Criteria for Reporting Qualitative Research
GCP	Good Clinical Practice
