# Supplementary material for: Nurses’ Perceptions of Communication in an Oncology Hospital Care: A Qualitative Study
Source: Healthcare (Basel). 2026 Jan 4;14(1):121. doi: 10.3390/healthcare14010121 (PMC12785592; doi:10.3390/healthcare14010121)
Supplement: Supplementary file 1 [file healthcare-14-00121-s001.zip › Supplementary File S1.pdf]

File S1: COREQ checklist

Consolidated criteria for reporting qualitative studies (COREQ): 32-item checklist

Tong A, Sainsbury P, Craig J. Consolidated criteria for reporting qualitative research (COREQ): a 32-item checklist for interviews and focus groups. International Journal for Quality in Health Care. 2007. Volume 19, Number 6: pp. 349 – 357

**Item No**	**Guide Questions/Description**	**Reported on Page #**
**Domain 1: Research team and reflexivity**		
**Personal Characteristics**		
1. Interviewer/ facilitator	Which author/s conducted the interview?	Two researchers carried out the interviews (GDA and AC).
2. Credentials	What were the researcher's credentials? E.g., PhD, MD	Both GDA and AC has a Master's degree in Psychology
3. Occupation	What was their occupation at the time of the study?	Both GDA and AC were psycho-oncologists.
4. Gender	Was the researcher male or female?	Females.
5. Experience and training	What experience or training did the researcher have?	Both had received training in qualitative research methodologies and had previous experience using this methodology.
**Relationship with participants**		
6. Relationship established	Was a relationship established prior to study commencement?	No prior relationship was established between the researchers and participants.
7. Participant knowledge of the interviewer	What did the participants know about the researcher? E.g., personal goals, reasons for doing the research?	Participants knew where the researchers worked and the purpose of the research.
8. Interviewer characteristics	What characteristics were reported about the interviewer/facilitator? E.g., Bias, assumptions, reasons and interests in the research topic	Both GDA and AC were psychologists working in an oncology setting and had a strong interest in the subject.
**Domain 2: study design**		
**Theoretical framework**		

Item No	Guide Questions/Description	Reported on Page #
9. Methodological orientation and Theory	What methodological orientation was stated to underpin the study? E.g., grounded theory, discourse analysis, ethnography, phenomenology, content analysis	A qualitative descriptive, inductive and monocentric approach was chosen. The Framework Analysis approach was used for data analysis.
**Participant selection**		
10. Sampling	How were participants selected? E.g., purposive, convenience, consecutive, snowball	A purposive sample of oncology nurses had been recruited.
11. Method of approach	How were participants approached? E.g., face-to-face, telephone, mail, email	Face-to-face semi-structured interviews guided by key questions.
12. Sample size	How many participants were in the study?	A total of twenty nurses were recruited.
13. Non-participation Setting	How many people refused to participate or dropped out? Reasons?	Only 1 nurse did not agree to participate in the study because he was engaged in activities related to his professional work.
14. Setting of data collection	Where was the data collected? E.g., home, clinic, workplace	The interviews took place in the hospital, in a dedicated room, ensuring an uninterrupted environment and ensuring comfort and privacy for the participants.
15. Presence of nonparticipants	Was anyone else present besides the participants and researchers?	No. Only interviewers and interviewed nurses were present.
16. Description of sample	What are the important characteristics of the sample? E.g. demographic data, date	Participants completed a socio-demographic data form (age, gender, education level, date of employment, date of employment in oncology).
**Data collection**		
17. Interview guide	Were questions, prompts, and guides provided by the authors? Was it pilot tested?	The semi-structured questions were developed by the research team based on information obtained from a literature review, were submitted to expert judgement and then tested on 6 patients.
18. Repeat interviews	Were repeat interviews carried out? If yes, how many?	No.

Item No	Guide Questions/Description	Reported on Page #
19. Audio/visual recording	Did the research use audio or visual recording to collect the data?	Interviews were audio-recorded.
20. Field notes	Were field notes made during and/or after the interview or focus group?	No.
21. Duration	What was the duration of the interviews or focus group?	The average length of the interviews was 30 minutes.
22. Data saturation	Was data saturation discussed?	Data saturation was briefly described in the "Data collection" section.
23. Transcripts returned	Were transcripts returned to participants for comment and/or correction?	Transcripts were not returned to participants
**Domain 3: analysis and findings**		
**Data analysis**		
24. Number of data coders	How many data coders coded the data?	Data was coded and categorized independently by three researchers, while other researchers verified the consistency of these codes, categories, subthemes, and themes.
25. Description of the coding tree	Did the authors provide a description of the coding tree?	An example of the coding process in the inductive analysis was provided in Table 2 and the "Data Analysis" section.
26. Derivation of themes	Were themes identified in advance or derived from the data?	Themes were derived from the data.
27. Software	What software, if applicable, was used to manage the data?	NVivo
28. Participant checking	Did participants provide feedback on the findings?	No.
**Reporting**		
29. Quotations presented	Were participant quotations presented to illustrate the themes/findings? Was each quotation identified? E.g., participant number	Quotations have been presented in the results section, with participant codes assigned to all participants and used against quotations.

Item No	Guide Questions/Description	Reported on Page #
30. Data and findings consistent	Was there consistency between the data presented and the findings?	We endeavoured to report the study findings in a clear, consistent manner to accurately reflect the data that have been collected
31. Clarity of major themes	Were major themes clearly presented in the findings?	Yes, major themes are clearly presented in the results section.
32. Clarity of minor themes	Is there a description of diverse cases or a discussion of minor themes?	We committed to discussing all the data that emerged from the interviews.
