# Supplementary material for: Nurses’ Perceptions of Communication in an Oncology Hospital Care: A Qualitative Study
Source: Healthcare (Basel). 2026 Jan 4;14(1):121. doi: 10.3390/healthcare14010121 (PMC12785592; doi:10.3390/healthcare14010121)
Supplement: Supplementary file 1 [file healthcare-14-00121-s001.zip › Supplementary File S2.pdf]

File S2-Full Interview Guide

Note: This interview guide was originally developed in Italian and used during data collection. For publication, it has been translated into English through a two-step process involving translation by a bilingual researcher and independent review for semantic and conceptual accuracy.

Interview Guide (Semi-Structured, Phenomenological Approach)

Section 1 – Opening Questions

1. **Can you briefly describe your professional role and experience in oncology nursing?**
2. **How would you describe your general approach to communicating with patients in your daily practice?**

Section 2 – Core Narrative Questions (Phenomenological Focus)

1. **Can you recall a recent situation in which communication with a patient was particularly meaningful or challenging?**
    - *Probe:* What made this situation significant for you?
    - *Probe:* How did you feel during this interaction?
    - *Probe:* What was the patient's reaction?
2. **In your experience, what factors facilitate effective communication with oncology patients?**
    - *Probe:* Can you describe a concrete episode where communication worked well?
    - *Probe:* What elements helped you in that situation?
3. **What factors hinder communication with patients in your daily practice?**
    - *Probe:* Can you recall a specific moment when communication was difficult?
    - *Probe:* How did you manage the situation?
    - *Probe:* What would have helped?
4. **How do contextual or organizational aspects influence your communication with patients?**
    - *Probe:* Workload, environment, time constraints
    - *Probe:* Team dynamics or institutional procedures

Section 3 – Communication-Specific Exploration (RTA-Aligned Probes)

1. **How do you adapt your communication when patients experience emotional distress?**
   - *Probe:* What signs guide your approach?
   - *Probe:* What strategies do you find effective?
2. **How do you manage communication when delivering complex or sensitive information?**
   - *Probe:* How do you ensure clarity?
   - *Probe:* How do you support the patient's emotional response?
3. **How do you encourage patient participation in treatment decisions?**
   - *Probe:* What techniques help patients express their preferences?
   - *Probe:* What barriers do you encounter?

Section 4 – Closing Question

1. **Based on your experience, what practical suggestions would you offer to improve nurse–patient communication in oncology settings?**
2. **Is there anything else you would like to add regarding communication with patients?**

Section 5 – Socio-Demographic Form

Participants were asked to provide:

- Age;
- Gender;
- Years of professional experience;
- Years of experience in oncology;
- Current clinical unit;
- Educational background;
- Additional training in communication (if any).
